# Giant Cell Tumor of Soft Tissue: An Updated Review

**DOI:** 10.3390/jcm13102870

**Published:** 2024-05-13

**Authors:** Jun Nishio, Shizuhide Nakayama, Kaori Koga, Mikiko Aoki

**Affiliations:** 1 Section of Orthopaedic Surgery, Department of Medicine, Fukuoka Dental College, 2-15-1 Tamura, Sawara-ku, Fukuoka 814-0193, Japan; 2 Department of Orthopaedic Surgery, Faculty of Medicine, Fukuoka University, 7-45-1 Nanakuma, Jonan-ku, Fukuoka 814-0180, Japan; n.shizuhide@gmail.com; 3 Department of Pathology, Faculty of Medicine, Fukuoka University, 7-45-1 Nanakuma, Jonan-ku, Fukuoka 814-0180, Japan; kogakao@fukuoka-u.ac.jp (K.K.); mikikoss@fukuoka-u.ac.jp (M.A.)

**Keywords:** giant cell tumor of soft tissue, giant cell tumor of bone, diagnosis, pathogenesis, treatment, tenosynovial giant cell tumor, keratin-positive giant cell-rich tumor

## Abstract

Giant cell tumor of soft tissue (GCTST) is a locally aggressive mesenchymal neoplasm of intermediate malignancy that predominantly occurs in the superficial soft tissue of the extremities. It is histologically similar to a giant cell tumor of bone (GCTB) and shows a mixture of round to oval mononuclear cells and osteoclast-like multinucleated giant cells. Currently, immunohistochemistry plays a very limited role in the diagnosis of GCTST. Primary or secondary malignant GCTST has recently been described and tumors exhibiting high-grade histological features demonstrate higher rates of distant metastasis. GCTST lacks the *H3-3A* gene mutations that are identified in the vast majority of GCTBs, suggesting a different pathogenesis. Surgery is the standard treatment for localized GCTST. Incomplete surgical resection is usually followed by local recurrence. Radiation therapy may be considered when the close proximity of critical structures prevents microscopically negative surgical margins. The systemic treatment options for advanced or metastatic disease are very limited. This review provides an updated overview of the clinicoradiological features, pathogenesis, histopathology, and treatment for GCTST. In addition, we will discuss the differential diagnosis of this peculiar neoplasm.

## 1. Introduction

Giant cell tumor of soft tissue (GCTST) is an extremely rare, locally aggressive neoplasm first described by Salm and Sissons in 1972 [1]. It belongs to the so-called fibrohistiocytic tumor group, according to the latest World Health Organization classification of soft tissue and bone tumors [2]. The term is sometimes confused with tenosynovial giant cell tumor (TSGCT). The etiology of this neoplasm is unknown. GCTST is histologically similar to, but genetically different from, giant cell tumor of bone (GCTB) [3,4]. Clinically, GCTST has a tendency to recur locally. Although distant metastasis is rare, primary or secondary malignant GCTST has recently been reported [5,6,7,8,9,10]. Surgical resection remains the mainstay of treatment for localized GCTST, and modern imaging techniques may reduce the occurrence of incomplete resection. Long-term follow-up is recommended to monitor for potential local recurrence or metastasis. There is currently no consensus regarding the optimal treatment strategy for advanced or metastatic disease. In this article, we review the clinical, radiological, histological, and genomic features of GCTST, summarize the current management, and discuss the differential diagnosis of this ultra-rare entity.

## 2. Clinical Characteristics

GCTST has a peak incidence in the fifth decade of life (age range: 5–89 years) and shows no gender predilection [2]. It typically presents as a painless, superficial soft tissue mass. Deep soft tissue involvement can be seen [11]. The diameter ranges from 0.7 to 10 cm [5,11,12]. Approximately two-thirds of GCTSTs arise in the upper and lower extremities. GCTST may also occur in less common sites, such as the trunk and head and neck [12,13]. The duration of symptoms before diagnosis ranges from 2 to 12 months, with a mean duration of 6.1 months [12].

The biological behavior of GCTST is unpredictable. Local recurrences occur in 6.2–21% of cases [11,12]. Incomplete surgical resection with positive margins is associated with an increased risk of local recurrence. Distant metastases are rare in conventional GCTST (up to 6.2%) [12]. Oliveira et al. reported on one patient with conventional GCTST, who had multiple local recurrences and lung metastases and died of the tumor 12 months after initial treatment [12]. The lung appears to be the most common distant metastatic site for GCTST. No clinicopathological factors are currently predictive of metastatic behaviors associated with pure GCTST. In contrast, malignant GCTST possesses high metastatic potential [5,9,10]. O’Connell et al. reported that of four patients with malignant GCTST, one died of metastatic disease at 13 months and one developed local recurrence at 84 months [5]. Recently, Iwai et al. reported a case of malignant GCTST of the intrinsic back musculature, with both postoperative local recurrence and multiple lung metastases [9]. The patient eventually succumbed to the disease, in spite of systemic treatment. Most recently, Hata et al. reported a case of primary GCTST of the kidney and its malignant transformation due to peritoneal dissemination [10]. The patient developed multiple masses in the retroperitoneum 4 years and 5 months after primary surgery. Unfortunately, the patient died 6 months after the peritoneal resection. Based on these findings, we suggest that long-term follow-up may be necessary to monitor for any signs of local recurrence or metastasis.

## 3. Imaging Features

There is only limited description of the imaging appearance of GCTST. Radiographs may be normal or show a non-specific soft tissue mass. In such cases, it is often impossible to establish a meaningful differential diagnosis. Peripheral calcification of the tumor may be seen [14]. Although osseous erosion or invasion has been reported in only a few cases [8,14], the underlying bone is typically normal. Ultrasonography can confirm the presence of a suspected lesion and shows a variable appearance [14,15,16,17,18]. GCTST has been described as homogeneous, hypoechoic areas (cystic portions), or heterogeneous, more echoic areas (solid portions). Color Doppler examination may display increased vascularity [14,18]. Computed tomography (CT) shows a solid, heterogeneous, predominantly hypodense mass [8,18,19,20,21]. Contrast-enhanced CT demonstrates heterogeneous enhancement. On magnetic resonance imaging (MRI), GCTST usually shows a solid soft tissue mass with low to intermediate signal intensity on T1-weighted images and variable signal intensity on T2-weighted images [8,14,20]. On the other hand, An et al. reported one case of GCTST displaying a predominantly cystic mass [15]. Fluid–fluid levels, suggesting a secondary aneurysmal bone cyst (ABC) formation, can be seen [17,22]. Contrast-enhanced MRI demonstrates avid enhancement of cyst walls and solid portions. Technetiumc-99m methylene diphosphonate bone scintigraphy is rarely used in the diagnosis of GCTST, but may reveal increased activity of the lesion [8,23]. Positron emission tomography (PET) imaging has been reported in only limited cases and demonstrates increased fluorodeoxyglucose (FDG) uptake, with high standard uptake values [7,14,18]. Similarly, it has been recognized that giant cell-rich tumors, such as TSGCT and GCTB, have a high accumulation of FDG [24,25], resulting in false-positive diagnoses of malignancy on PET.

## 4. Pathogenesis

Only one case of GCTST has cytogenetically been characterized in the literature [26]. Like GCTB, cytogenetic analysis shows numerous telomeric associations involving multiple chromosomes.

In recent years, there has been an important breakthrough in the molecular profile of GCTB [27]. The vast majority of cases harbor H3.3 histone A (*H3-3A*) gene mutations [28,29,30]. *H3-3A*, located on 1q42, is one of the two genes encoding the histone H3 variant (H3.3). H3.3 has an essential function in maintaining genome integrity in development and the cell cycle incorporated in chromatin. Interestingly, the most frequent mutation (H3.3 p.Gly34Trp mutation) can readily be detected using immunohistochemistry (anti-histone H3.3 G34W rabbit monoclonal antibody) and its immunohistochemical analysis is a reliable and highly specific method for distinguishing GCTB from its histological mimics [31,32,33]. On the other hand, GCTST lacks *H3-3A* gene mutations [3,4]. Based on these findings, we strongly suggest that GCTST may be genetically different from GCTB.

Previous studies have shown that the neoplastic mononuclear stromal cells in GCTST are, at least in part, of osteoblastic lineage and express alkaline phosphatase (ALP), receptor activator of nuclear factor kappa B (RANK), runt-related transcription factor 2 (RUNX2), osteoprotegerin (OPG), and tumor necrosis factor-related apoptosis-inducing ligand (TRAIL) [4,34]. Compared to GCTB, GCTST reveals lower expression of receptor activator of nuclear factor kappa B ligand (RANKL) and SATB homeobox 2 (SATB2) [4]. On the other hand, the osteoclast-like giant cells in GCTST are positive for RANK, tartrate-resistant acid phosphatase (TRAP), and vitronectin receptor (VNR), but negative for RUNX2, RANKL, or SATB2 [4,34]. Currently, the precise role of RANKL pathway in osteoclast formation in GCTST is unknown.

In 2020, Rajaii et al. reported that next-generation sequencing revealed a splicing variant of partner and localizer of BRCA2 (*PALB2*) in one case of metastatic GCTST of the orbit [35]. *PALB2* is a tumor suppressor gene with a role in the homologous recombination repair pathway, through interaction with both BRCA1 DNA repair associated (*BRCA1*) and BRCA2 DNA repair associated (*BRCA2*) [36]. Homozygous pathogenetic variants of *PALB2* cause Fanconi anemia, while heterozygous constitutional (germline) pathogenic variants are associated with an increased risk of cancer, predominantly breast cancer [37].

Most recently, Hata et al. reported that a cancer multi-gene panel testing demonstrated mutations of phosphatidylinositol-4,5-bisphosphate 3-kinase catalytic subunit alpha (*PIK3CA*) and deletions of cyclin-dependent kinase inhibitor 2A/B (*CDKN2A/B*), methylthioadenosine phosphorylase (*MTAP*), and lysine demethylase 6A (*KDM6A*) in one case of GCTST, with malignant transformation [10]. Further studies are needed to elucidate the biological consequences of these genomic alterations in GCTST.

## 5. Histopathology

Grossly, GCTST is a well-circumscribed, mostly solid, nodular mass with a fleshy, red-brown or gray cut surface [2]. Gritty regions corresponding to mineralized bone are often present at the periphery of the tumor [5]. Hemorrhage and necrosis may be seen in cases of high-grade GCTST.

Histologically, most GCTSTs show a multinodular architecture, with cellular nodules separated by fibrous septa. The nodules are composed of a mixture of mononuclear cells and osteoclast-like multinucleated giant cells in a richly vascularized stroma [2]. The mononuclear cells have round to oval vesicular nuclei. Mitotic figures are found only in the mononuclear cells, with an average count of 2–3 mitoses per 10 high-power fields [5,11]. Nuclear pleomorphism and bizarre giant cells are absent, and necrosis is uncommon [2]. Metaplastic bone formation is present in approximately 40–50% of cases [11,12]. ABC-like changes (27%), stromal hemorrhage and hemosiderin deposition (50%), and clusters of foamy macrophages (68%) may be seen [12]. Moreover, vascular invasion is identified in approximately 32% of cases and does not appear to portend a poor prognosis, or to be associated with the presence of synchronous or metachronous metastatic disease [11,12].

In contrast to conventional GCTST, high-grade examples of GCTST demonstrate increased mitotic activity and nuclear pleomorphism [5]. In addition, atypical mitotic figures are common and necrosis is frequently found. Like conventional GCTSTs, however, a multinodular growth pattern is present in high-grade GCTSTs [5].

Immunohistochemically, the mononuclear cells are focally positive for CD68 and smooth muscle action (SMA). The osteoclast-like giant cells demonstrate strong and diffuse positive staining for CD68, but are negative for SMA. Immunostainings for desmin, CD31, CD45, lysozyme, and S-100 protein are typically negative. We speculate that immunohistochemistry does not play a significant role in the diagnosis of GCTST.

## 6. Management

### 6.1. Localized Disease

Surgical resection is the standard treatment for localized GCTST. The surgical procedure consists of a wide resection with negative margins (R0, no residual microscopic tumor) [12]. Reconstructive surgery may help to achieve a R0 surgery. Re-resection is recommended for patients with positive margins [5,11]. In general, limb-sparing and function-sparing approaches should be used, where feasible, for local disease of the extremities. In very selected cases, amputation might be an option when a wide resection fails to preserve limb function [38]. It has been suggested that excellent local control in low-grade soft tissue sarcoma (STS) may be achieved with microscopic margins greater than 2 mm [39]. Currently, an adequate margin of resection for GCTST is not well established, because of its infrequent occurrence and the limited number of reported cases.

Radiation therapy (RT) can be used as a perioperative treatment strategy to improve local disease control. The role of RT in the management of GCTST remains controversial. There are several case reports concerning the use of adjuvant RT in patients with conventional GCTST [16,21,40,41,42]. Short-term follow-up showed a satisfactory response in most patients. These results suggest that postoperative RT may be considered for cases with incomplete surgical margins, due to the close proximity of critical structures, particularly in the head and neck region [13]. On the other hand, recently, Chen et al. evaluated the treatment effectiveness of surgery alone, or surgery combined with RT (surgery + RT), in patients with malignant GCTST, using the Surveillance, Epidemiology, and End Results (SEER) database (1975–2016) [6]. In this retrospective study, the surgery alone group had a better 10-year survival profile than the surgery + RT group. In addition, a subsequent subgroup analysis demonstrated that the surgery alone group had a similar 10-year survival, compared with the surgery + RT group, for patients with high-grade histology and distant metastasis. The authors suggested that RT should not be recommended as a regular therapeutic method for patients with malignant GCTST. Further prospective randomized trials are required to better define optimal treatment approaches for localized GCTST.

### 6.2. Advanced/Metastatic Disease

The development of unresectable, locally advanced, or metastatic GCTST is associated with a worse prognosis [5,9,10]. Currently, there is no regulatory approved treatment for advanced/metastatic GCTST.

Pazopanib is an oral multi-target tyrosine kinase inhibitor (TKI) with anti-angiogenic and anti-tumorigenic properties, which has been approved in multiple countries as a second- or later-line treatment for patients with advanced STS. Recently, Iwai et al. reported the use of pazopanib in the treatment of metastatic malignant GCTST [9]. In this case, the patient developed local recurrence and multiple lung metastases 3 months after surgical resection and was immediately treated with pazopanib 400 mg once daily. Regression of lung metastases was observed during the first month of treatment with pazopanib, without severe adverse effects. The authors concluded that pazopanib treatment for metastatic GCTST was effective and its efficacy lasted for 11 months. However, the role of pazopanib in advanced/metastatic GCTST remains unclear and requires further investigation in a randomized clinical trial.

Lenvatinib is a small-molecule TKI that inhibits vascular endothelial growth factor receptor (VEGFR) 1-3, fibroblast growth factor receptor (FGFR) 1-4, platelet-derived growth factor receptor alpha (PDGFRα), KIT proto-oncogene, receptor tyrosine kinase (KIT), and is rearranged during transfection (RET). Recent evidence indicated a promising role of lenvatinib, in monoregimen or in combination, for the management of GCTB [43]. In addition, a multicenter, open-label, multicohort phase 1/2 trial has highlighted lenvatinib in combination with etoposide plus ifosfamide, as a promising antitumor drug in patients with refractory or relapsed osteosarcoma [44]. Moreover, clinical trials of lenvatinib in combination with pembrolizumab are currently ongoing in selected advanced/metastatic STS and bone sarcomas, including osteosarcoma and chondrosarcoma (NCT04784247).

Zoledronic acid is a bisphosphonate that has recently gained interest in adjuvant therapy for GCTB. Several in vitro and in vivo studies have shown that zoledronic acid induces proliferation inhibition and apoptosis of neoplastic stromal cells and osteogenic differentiation [45,46,47,48,49]. Recently, a multicenter randomized open-label phase II trial was performed to compare the local recurrence rate after surgery, between the adjuvant zoledronic acid group (*n* = 8) and the control group (*n* = 6), for high-risk GCTB [50]. During a median follow-up of 93.5 months, the 2-year recurrence rates were 38% (3/8) in the adjuvant zoledronic acid group and 17% (1/6) in the control group (*p* = 0.58). All recurrences were seen within the first 15 months after surgery. The authors concluded that adjuvant zoledronic acid treatment for high-risk GCTB did not decrease the local recurrence rate. In contrast, another prospective, randomized trial, with neoadjuvant zoledronic acid combined with surgery (*n* = 15) versus surgery alone (*n* = 15), was performed to assess the clinical, radiological, and histopathological effects of zoledronic acid in patients with GCTB of the extremities [51]. Pain was reduced and follow-up radiographs showed increased mineralization at the periphery of the lesion in the neoadjuvant zoledronic acid combined with surgery group. In addition, the neoplastic cells had a significantly higher apoptosis index after administration of zoledronic acid. The authors stated that zoledronic acid treatment succeeded in controlling tumor growth. There are two case reports concerning the use of zoledronic acid in cases of unresectable GCTST [52,53]. Mokrani et al. reported a case of primary GCTST of the elbow that was judged unresectable due to its intra-articular extension [52]. The patient received zoledronic acid injections for 8 months and had stable disease. Nepucpan et al. also reported a case of primary GCTST of the nasopharynx that was regarded as unresectable due to its location [53]. Zoledronic acid was administered once every 28 days for three cycles as a first-line systemic treatment. Although follow-up CT revealed stable disease after three cycles of zoledronic acid, the patient reported new-onset or persisting symptoms, therefore a decision was made to shift to denosumab. These results should be interpreted with caution, because the number of cases is too small to make any definite conclusion.

After its United States Food and Drug Administration (FDA) approval, denosumab, a human monoclonal IgG2 antibody, is widely used to treat unresectable GCTB in adults and skeletally mature adolescents [54,55,56]. It acts by binding to and inhibiting RANKL. A recent randomized clinical trial revealed that denosumab was more effective and safer than zoledronic acid for the treatment of unresectable GCTB [57]. Similarly to GCTB, GCTSTs also have a histological presence of osteoclast-like giant cells and express high levels of RANK/RANKL. Therefore, it is hypothesized that GCTST will show the same response to denosumab as previously seen in GCTB. There are three case reports of denosumab treatment in patients with advanced GCTST [18,35,53]. Chen et al. described a good response to denosumab treatment in a 70-year-old woman with advanced GCTST of the thyroid [18]. Six months after revision surgery, the patient developed multiple local recurrences and suffered from dysphagia and breathlessness. The patient received subcutaneous injections of denosumab 120 mg every 28 days, with an additional loading dose on days 8 and 15 of the first month. Three months after initiation of denosumab, the patient demonstrated symptom relief and tumor regression. The patient continued to have tumor regression after one year of the denosumab treatment. As noted above, Nepucpan et al. reported the use of denosumab in a 30-year-old man with advanced GCTST [53]. After zoledronic acid treatment, denosumab was administered every month for three cycles, with an additional loading dose on days 8 and 15 of the first month. Three months after denosumab treatment, the patient showed symptom improvement and stable disease. Rajaii et al. also reported the use of denosumab in a 34-year-old woman with advanced GCTST of the orbit [35]. Four months after primary surgical resection, the patient developed local recurrence and had revision surgery. The patient received adjuvant denosumab therapy (three doses); however, 4 months later, the patient developed local recurrence with intracranial extension. Clinical benefits were not observed following denosumab treatment. For the related group of giant cell-rich tumors, including GCTST, the place of denosumab is still up for further investigation [58].

Immunotherapy is an emerging treatment for several cancer types, with promising outcomes. The major targets of FDA-approved immunotherapeutic antibodies are programmed cell death protein-1 (PD-1) and its ligand-programmed cell death, ligand-1 (PD-L1). There are few studies concerning the expression of PD-L1 in GCTB [59,60]. An immunohistochemical study indicated PD-L1 expression by neoplastic cells and osteoclast-like giant cells in 28.3% of GCTB cases and PD-L1 expression was associated with shorter disease-free survival (59]. Another immunohistochemical study also showed that PD-L1 expression was observed in about 28% of primary GCTB cases without denosumab treatment and PD-L expression was significantly correlated to shorter recurrence-free survival [60]. These findings suggest that PD-L1 may be a potential new therapy target in GCTB. However, there are currently no studies concerning tumor immunity in GCTSTs.

## 7. Differential Diagnosis

The differential diagnosis for GCTST is broad and should include benign and malignant giant cell-rich tumors. In our experience, GCTST is most often confused with TSGCT. Moreover, keratin-positive giant cell-rich tumor of soft tissue (KPGCTST) can be challenging to distinguish from GCTST, especially in small specimens. The corresponding clinicopathological and molecular characteristics are summarized in Table 1.

TSGCT, formerly known as giant cell tumor of tendon sheath or pigmented villonodular synovitis, is a mesenchymal neoplasm that most often arises from the synovium of joints, bursae, and tendon sheaths. TSGCT is classified into localized and diffuse types, based on its growth pattern. Malignant TSGCT is exceedingly uncommon and can arise de novo or following multiple recurrences of conventional TSGCT. Localized TSGCT presents as a non-destructive, well-circumscribed mass or nodule that predominantly occurs in the fingers. In contrast, diffuse-type TSGCT is defined as a poorly circumscribed, infiltrative soft tissue mass that usually occurs in large joints, particularly the knee. TSGCT affects all age groups, but usually young and middle-aged adults, with a slight female predominance [61]. Surgery is the mainstay of the treatment of TSGCT when it can be accomplished without significant morbidity [62]. Local recurrences are more common in diffuse-type TSGCT, occuring in 40–60% of cases [61]. Due to the high recurrence rate, patients will go through multiple surgical resections. Histologically, TSGCT consists of a mixture of mononuclear cells and osteoclast-like multinucleated giant cells. There are two types of mononuclear cells: small histiocyte-like cells and larger epithelioid cells. Compared with GCTST, TSGCT has prominent stromal collagenization and a more heterogeneous population of cells, including foamy macrophages and inflammatory cells [11]. Moreover, unlike GCTST, metaplastic bone formation is extremely rare in TSGCT. Expression of clusterin is found in the large mononuclear cells [63]. As mentioned above, GCTST is negative for desmin, whereas desmin-positive cells are seen in 43% of TSGCT cases [64]. Cytogenetic studies have revealed recurrent rearrangements of 1p13 in a subpopulation of TSGCTs [65,66,67,68]. In recent years, there has been a remarkable breakthrough in the molecular profile of TSGCT. TSGCT is caused by upregulation of the colony-stimulating factor 1 (*CSF1*) gene, resulting in aberrant CSF1 expression and recruitment of CSF1 receptor (CSF1R)-dependent inflammatory cells to the affected joints [69]. Therefore, inhibition of signaling between CSF1 and CSF1R targets the underlying cause of the disease [70]. In 2019, the FDA approved pexidartinib for adult patients with symptomatic TSGCT associated with severe morbidity, or functional limitations not amenable to improvement with surgery [62]. Pexidartinib is an oral tyrosine kinase inhibitor with selective inhabitation of CSF1R. To the best of our knowledge, aberrant expression of CSF1 has not been identified in GCTST. Considering these findings, however, we speculate that CSF1R inhibitors such as pexidartinib might be a possible therapeutic option for advanced/metastatic GCTST, if the CSF1/CSF1R pathway is involved in the pathogenesis of GCTST.

KPGCT is an extremely rare, recently described mesenchymal neoplasm which can affect both soft tissue and bone [71,72,73,74,75,76]. In 2021, Agaimy et al. described a series of six cases similar to GCTST, but with a subpopulation of keratin-positive cells and a novel high mobility group AT-hook 2 (*HMGA2*)-nuclear receptor corepressor 2 (*NCOR2*) fusion [71]. The authors proposed the term “KPGCTST with *HMGA2*-*NCOR2* fusion” for this novel entity. In 2022, Dehner et al. reported a series of nine cases showing overlapping morphological features of KPGCT and xanthogranulomatous epithelial tumor (XGET) [72]. The *HMGA2*-*NCOR2* fusion was detected in 7 out of 9 cases. The authors suggested that these two tumors may represent morphological variants of a single entity. Compared with GCTST, KPGCTST shows a distinct predilection for female and a younger age demographic (a median age of 23 years) [75]. KPGCTST presents as a superficial soft tissue mass of variable size, which frequently occurs in the extremities. Due to the limited number of reported cases, the optimal management of KPGCTST is yet to be established. Surgical resection appears to be the primary treatment modality [71,74,75]. The majority of cases show no evidence of disease at follow-up, but available data are unfortunately limited. One patient described in the literature had local recurrence 8 months after the initial fragmented resection and had been disease-free for 16 months after re-resection with negative margins [74]. Distant metastasis has not been reported to date. Histologically, KPGCTST is a uninodular mass composed of sheets of mononuclear cells with admixed evenly distributed osteoclast-like multinucleated giant cells and variably lobulated architecture. A variably intense lymphocytic infiltrate at the periphery of the lesion can be seen [71]. Necrosis, stromal hemorrhage, and hemosiderin deposition may be observed [71]. Although it may be difficult to distinguish between KPGCTST and GCTST only on the basis of morphology, the lack of metaplastic bone formation and the presence of perilesional lymphoid reaction are clues suggestive of a diagnosis of KPGCTST. Moreover, unlike GCTST, KPGCTST is positive for keratin in the mononuclear neoplastic cells. Endothelial, myoid, and neurogenic markers are typically negative [71]. It is of interest that *CSF1* expression, similarly to TSGCT, has also been identified in KPGCTST [74,77]. In 2023, Perret et al. reported that whole RNA-sequencing revealed similar levels of expression of the CSF1/CSF1R axis between KPGCTST and TSGCT [74]. Most recently, Dehner et al. also identified strong expression of *CSF1* mRNA and high-level *CSF1* gene expression in KPGCTST/XGET [77]. These findings show that the CSF1/CSF1R pathway is involved in the pathogenesis of KPGCTST. These findings also indicate the possibility that CSF1R inhibitors may have efficacy in the treatment of patients with advanced/metastatic KPGCTST.

Undifferentiated pleomorphic sarcoma (UPS) with giant cells, formerly known as giant cell malignant fibrous histiocytoma, may be considered in the differential diagnosis of GCTST, particularly in malignant variants [12]. UPS is currently defined as an exclusive neoplasm that shows no identifiable line of differentiation [78]. Although UPS can affect all age groups, it mostly occurs in older adults. Unlike GCTST, UPS most frequently arises in the deep soft tissue of the extremities. The majority of UPSs are morphologically high grade. In general, UPS is cytogenetically characterized by complex chromosome aberrations [79]. Surgery is the primary treatment modality and is often supplemented with RT and chemotherapy [80]. Immunotherapy may be of benefit in a subset of patients with advanced or metastatic UPS [80]. Local recurrences occur in 22.9% of cases [81]. The 5-year metastasis-free survival rate is 70% [82]. Histologically, UPS exhibits atypical, pleomorphic spindle cells with abundant mitotic figures. Notably, approximately 10–15% of cases show the presence of multinucleated osteoclast-like giant cells [83]. Recently, Matsuoka et al. reported one case of malignant GCTST of the chest wall that was initially diagnosed as UPS with giant cells [84]. This case showed aggressive clinical features and was reclassified as malignant GCTST because the immunohistochemical examination demonstrated expression of RUNX2 and SATB2, displaying osteogenic differentiation. These findings indicate that the diagnosis of UPS with giant cells can be made only if no specific line of differentiation is identified.

**Figure 1:**
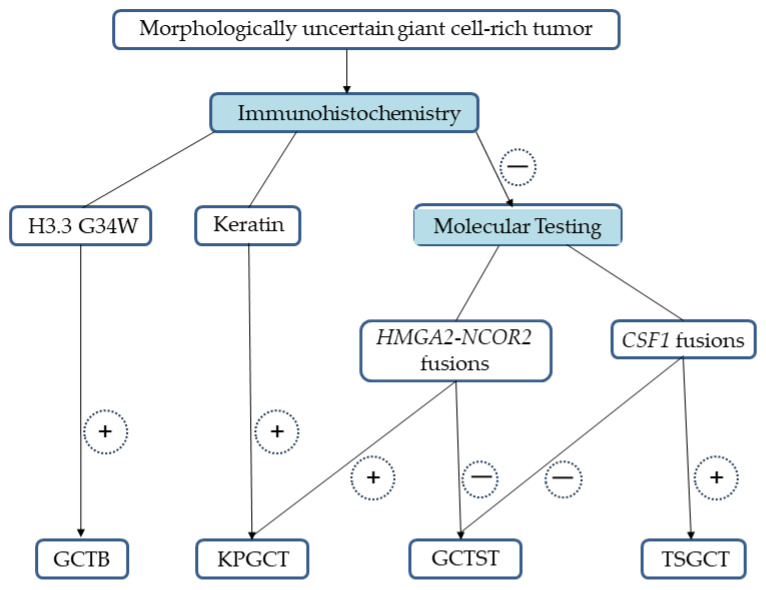
Proposed diagnostic algorithm for morphologically uncertain giant cell-rich tumors. GCTB: giant cell tumor of bone; KPGCT: keratin-positive giant cell-rich tumor, GCTST: giant cell tumor of soft tissue; TSGCT: tenosynovial giant cell tumor; *HMGA2*: high mobility group AT-hook 2; *NCOR2*: nuclear receptor corepressor 2; *CSF1*: colony-stimulating factor 1.

We propose a diagnostic algorithm for morphologically uncertain giant cell-rich tumors (Figure 1). Immunohistochemistry with H3.3 G34W and keratin is recommended as a starting point for the detection of underlying GCTB and KPGCT, respectively. In immunohistochemically ambiguous cases, molecular testing of *HMGA2*-*NCOR2* fusions can be used to distinguish KPGCT from its histological mimics, including GCTST. Moreover, the detection of *CSF1* fusions and/or *CSF1* rearrangements would be useful for the diagnosis of TSGCT, especially in limited tissue samples.

## 8. Conclusions and Future Directions

GCTST is an ultra-rare soft tissue tumor, with recurrent but minimal metastatic potential that predilects the superficial soft tissues of the extremities in middle-aged adults. High-grade GCTST has a greater risk for local recurrence and distant metastasis. Histologically, GCTST is similar to GCTB, and consists of round/oval mononuclear cells and osteoclast-like multinucleated giant cells. Immunohistochemistry plays a very limited diagnostic role and GCTST lacks *H3-3A* gene mutations. Surgical resection is the mainstay of treatment for localized GCTST. Adjuvant RT may be considered for cases with positive/close surgical margins. In the clinical practice setting, the therapeutic management of advanced/metastatic GCTST remains challenging. Denosumab may be a promising treatment option for selected patients with advanced disease, although lifelong treatment is not desirable. A better understanding of the biological and molecular characteristics of GCTST is required to develop novel therapies able to efficiently target specific pathways. It is often difficult to conduct robust clinical trials in ultra-rare tumors like GCTST; therefore, sustainable global and regional collaborative efforts are needed to improve the clinical outcomes of advanced/metastatic GCTST in the future.

## Figures and Tables

**Table 1:** Differential diagnosis of GCTST.

Entity	Age/Gender	Clinical Features	Histopathological Features	Molecular Features
GCTST	Fifth decade, equal male and female incidence.	Locally aggressive and rarely metastasizing; most cases occur in the superficial soft tissue of the extremities.	Multinodular lesion; composed of mononuclear cells and osteoclast-like giant cells. Metaplastic bone formation is present.	Limited data, no *H3-3A* gene mutations.
TSGCT	Young and middle-aged adults, slight female predominance.	Varied potential for local recurrence; LTSGCT predominantly occurs in the fingers and DTSGCT often occurs in the knee.	LTSGCT: well-circumscribed lesion; DTSGCT: poorly circumscribed lesion, composed of small histiocyte-like cells, larger epithelioid cells, and osteoclast-like giant cells. Fomay macrophages and desmin-positive cells can be seen.	*CSF1* fusions (usually with *COL6A3)*.
KPGCT	Limited data: median age of 23 years, female predominance.	The majority of cases show no evidence of disease at follow-up and occur in the superficial soft tissue of the extremities.	Uninodular lesion, composed of keratin-positive mononuclear cells and evenly distributed osteoclast-like giant cells. A mixed inflammatory infiltrate is present at the periphery.	*HMGA2*-*NCOR2* fusions.
UPS	Older adults, no distinct gender predominance.	Local recurrence and metastasis are common, most cases occur in the deep soft tissue of the extremities.	Infiltrative lesion; composed of atypical, pleomorphic cells with abundant mitotic figures. Osteoclast-like multinucleated giant cells are seen in 10–15% of cases.	Complex, no recurrent aberrations.

GCTST: giant cell tumor of soft tissue; TSGCT: tenosynovial giant cell tumor; KPGCT: keratin-positive giant cell-rich tumor; UPS: undifferentiated pleomorphic sarcoma; LTSGCT: localized tenosynovial giant cell tumor; DTSGCT; diffuse-type tenosynovial giant cell tumor; *H3-3A*: H3.3 histone A; *CSF1*: colony-stimulating factor 1; *COL6A3*; collagen type VI alpha 3 chain; *HMGA2*: high mobility group AT-hook 2; *NCOR2*: nuclear receptor corepressor 2.

## Data Availability

Not applicable.
